# Partial protection against collagen antibody-induced arthritis in PARP-1 deficient mice

**DOI:** 10.1186/ar1865

**Published:** 2005-12-06

**Authors:** Samuel García, Ana Bodaño, Antonio González, Jerónimo Forteza, Juan J Gómez-Reino, Carmen Conde

**Affiliations:** 1 Research Laboratory, Hospital Clínico Universitario, Choupana s/n, 15706-Santiago de Compostela, Spain; 2 Department of Pathology, Hospital Clínico Universitario, Choupana s/n, 15706-Santiago de Compostela, Spain; 3 Rheumatology Unit, Hospital Clínico Universitario and Department of Medicine, Universidad de Santiago, San Francisco s/n, 15700-Santiago de Compostela, Spain

## Abstract

Poly(ADP-ribose) polymerase-1 (PARP-1) is a nuclear DNA-binding protein that participates in the regulation of DNA repair and maintenance of genomic integrity. In addition, PARP-1 has a role in several models of inflammation disease, where its absence or inactivation confers protection. The aim of this study was to analyze the impact of selective PARP-1 suppression in collagen antibody-induced arthritis. We show that PARP-1 deficiency partially reduces the severity of arthritis, although the incidence of disease was similar in control and deficient mice. Decreased clinical scores were accompanied by partial reduction of histopathological findings. Interestingly, quantitative real-time PCR and ELISA analysis revealed that the absence of PARP-1 down-regulated IL-1β and monocyte chemotactic protein 1 expression in arthritic joints whereas tumor necrosis factor-α transcription was not impaired. Our results provide evidence of the contribution of PARP-1 to the progression of arthritis and identify this protein as a potential therapeutic target for the treatment of rheumatoid arthritis.

## Introduction

Rheumatoid arthritis (RA) is characterized by inflammation, synovial hyperplasia, pannus formation and progressive destruction of cartilage and bone [1,2]. In RA, inflammatory cytokines, chemokines, growth factors and adhesion molecules are produced by leukocytes and resident synoviocytes. These factors perpetuate chronic inflammation by the recruitment of additional inflammatory cells into the sublining region that, in turn, lead to continuous production of inflammatory mediators and enzymes, resulting in destruction of joint structures [3-8]. The efficacy of treatments with tumor necrosis factor (TNF) and IL-1 inhibitors strongly support the key role of inflammatory cytokines in the pathogenesis of RA [9,10] and points to therapeutic approaches directed toward regulation of cytokine networks involved in RA.

Poly(ADP-ribose) polymerase (PARP)-1 is a highly conserved nuclear zinc-finger protein involved in maintenance of genomic integrity. PARP-1 detects DNA breakage generated by several genotoxic agents and synthesizes and transfers ADP ribose units (poly(ADPribosyl)ation activity) into acceptor proteins involved in the conservation of chromatin structure and DNA metabolism, modulating in this way DNA repair and cell survival [11,12]. Studies with PARP-1 deficient mice or with chemical inhibitors have enlarged the physiological role of this protein. In these situations, lack of PARP-1 function protects against several disorders with an inflammatory component, such as endotoxic shock [13], streptozotocin induced diabetes [14], chronic colitis [15] and uveitis [16]. Two mechanisms have been proposed to explain the role of PARP-1 in these diseases. One mechanism is related to massive PARP-1 activation induced by genotoxic injury developed during the inflammatory process. In this case, hyperactivated PARP-1 would lead to ATP depletion and cell dysfunction [17]. The other proposed mechanism is related to a functional link between PARP-1 and inflammation-related transcription factors. Several *in vivo *and *in vitro *studies have demonstrated the involvement of PARP-1 in the transcriptional activation of nuclear factor (NF)κB [13,18,19], but the proposed mechanisms are contradictory. There is evidence for mechanisms that are both dependent on and independent of auto-poly(ADP-ribosyl)ation function. In the first case, NFκB would be blocked by binding to PARP-1 and this union would be disrupted by PARP-1 auto-poly(ADP-ribosyl)ation [18]. In the second case, PARP-1 would act as a transcriptional co-activator in the binding of NFκB with its target DNA sequences [19]. Recently, it has also been reported that PARP-1 regulates other transcription factors implicated in stress/inflammation, such as AP-1, Oct-1, SP-1, YY-1 and Stat-1 [20,21]. Thus, in addition to its involvement in genome surveillance, PARP-1 appears to have a key role in inflammatory responses.

Here we report the impact of selective PARP-1 suppression on the collagen antibody-induced arthritis model (CAIA). This model, induced by passive immunization of mice with anti-type II collagen antibodies, allows the study of the effector phase of arthritis, where PARP-1 might be involved. We have found that the absence of PARP-1 partially reduced the severity of arthritis, likely by the impairment of IL-1β and monocyte chemotactic protein (MCP)-1 transcription in arthritic tissue. These results provide support for the contribution of PARP-1 in the progression of arthritis and open the possibility that specific inhibitors might become therapeutic tools in RA.

## Materials and methods

### Mice

Mice lacking PARP-1 (kindly provided by G de Murcia, CNRS, Strasbourg, France) have been described previously [22]. The mice used in these experiments were of mixed (C57BL/6 × 129Sv) background. More than ten different breeding pairs of *parp-1*^+/o ^mice were intercrossed to generate *parp-1*^+/+^, *parp-1*^+/o ^and *parp-1*^o/o ^mice. *Parp-1*^o/o ^mice and control matched littermates (*parp-1*^+/+^, *parp-1*^+/o^) were analyzed.

Genotypes were assessed by PCR of tail DNA. The mice were maintained in the mouse facility of the Facultad de Medicina de Santiago de Compostela. Animal care was in compliance with Spanish regulations on the protection of animals used for experimental and other scientific purposes (Real Decreto 223/1998). The experimental protocols were approved by the Animal Care and Use Committee of the University of Santiago de Compostela.

### Collagen antibody-induced arthritis (CAIA) and clinical scoring

CAIA was induced in 6-week-old male and female mice by intravenous injection on day 0 of 3 mg/mouse of an arthritogenic cocktail of 4 monoclonal anti-type II collagen antibodies (Arthrogen, Chondrex, Redmond, WA, USA) [23]. On day 2, mice were boosted with 50 μg of lipopolysaccharide by intraperitoneal injection. Arthritis was assessed every other day by two blinded observers until day 12, using a semi-quantitative clinical score ranging from 0 to 4: 0, no swelling; 1, slight swelling and erythema of the ankle, wrist or digits; 2, moderate swelling and erythema; 3, severe swelling and erythema; and 4, maximal inflammation with joint rigidity. The maximum possible score was 16 per mouse.

### Histological analysis

Hind limbs were prepared for histology by dissecting the skin and muscle, and then sectioning knee joints. Specimens were fixed for 24 hours and demineralized in phosphate-buffered saline-0.5 M EDTA for 10 days. Knee joints were embedded in paraffin and sections were cut and stained with hematoxylin and eosin (H&E) for evaluation of inflammation. For analysis of damage to cartilage, knee sections were stained with Toluidine blue, Safranin-O and Masson trichrome following standard methodology. The sections were scored by two blinded observers. Synovial inflammation was scored on a scale of 0 to 3: 0, no inflammation; 1, slight thickening of synovial cell layer and/or some inflammatory cells in the sublining; 2, thickening of synovial lining, infiltration of the sublining; and 3, pannus formation.

Exudate was scored according to the following scale: 0, no detectable neutrophil infiltration in the synovial space; 1, mild infiltration; 2, moderate infiltration; and 3, severe infiltration.

Cartilage damage was evaluated following a scale of 0 to 3: 0, normal cartilage; 1, cartilage surface irregularities and loss of metachromasia adjacent to superficial chondrocytes; 2, fibrillation of cartilage and formation of some chondrocyte clusters, with minor loss of surface cartilage; and 3, gross cartilage abnormalities, including loss of superficial cartilage, extension of fissures close to subchondral bone, and a large number of chondrocyte clusters.

### Fibroblast like synoviocytes

Fibroblast-like synoviocytes (FLSs) were isolated from *parp-1*^+/+^, *parp-1*^+/o ^and *parp-1*^o/o ^mice. Synovial tissue was minced and incubated with 1 mg/ml collagenase in serum-free DMEM (Gibco, Invitrogen, Barcelona, Spain) for 3 hours at 37°C. After digestion, FLSs were filtered trough a nylon cell strainer (BD Falcon, Franklin Lakes, NJ, USA), washed extensively, and cultured in DMEM supplemented with 10% v/v FCS (Gibco, Invitrogen), penicillin, streptomycin, and L-glutamine (Sigma, St Louis, MO, USA) in a humidified 5% CO_2 _atmosphere. After overnight culture, non-adherent cells were removed, and adherent cells were cultured in DMEM supplemented with 10% v/v FCS.

### Western blot analysis

Total proteins (20 μg) were separated by 10% SDS-PAGE, transferred to a PVDF membrane (Hybond-P, Amersham Biosciences, Buckinghamshire, UK) and probed with anti-PARP-1 (VIC-5, kindly provided by G de Murcia, CNRS, Strasbourg, France) and anti-actin (Sigma) antibodies as previously described [24]. Bound antibody was revealed with goat anti-rabbit-horseradish peroxidase (Rockland Immunochemicals Inc., Gilbertsville, PA, USA) and the blot was developed using the ECL plus detection system (Amersham Biosciences).

### Quantitative reverse transcription-PCR

Total RNA was obtained from joints of *parp-1*^+/+ ^and *parp-1*^o/o ^mice on day 7 following Arthrogen injection, and from joints of *parp-1*^+/+ ^and *parp-1*^o/o ^control mice without arthritis. We used the RNeasy Kit and RNase-Free DNase Set (Qiagen GmbH, Hilden, Germany) according to the manufacturer's instructions. One microgram of total RNA was subjected to cDNA synthesis using M-MLV reverse transcriptase, random primers and RNaseOUT recombinant ribonuclease inhibitor (Invitrogen). Quantitative real-time PCR was performed in duplicate in a Chromo-4 real-time thermal cycler (MJ Research, Waltham, MA, USA), using a LightCycler DNA Master SYBR Green I kit (Roche Diagnostics, Barcelona, Spain), according to the manufacturers' protocols. The specific primers used in these reactions are listed in Table 1. Relative levels of gene expression were normalized to the β-actin gene using the comparative Ct method, where Ct is the cycle at which the amplification is initially detected. The relative amount of mRNA from the different genes was calculated using the formula 2^-ΔΔCt^, where:

ΔΔCt = [Ct_target _- Ct_β-actin_]_WT or KO with arthritis _- [Ct_target _- Ct_β-actin_]_WT or KO controls_

For wild-type (WT) and PARP-1 deficient samples without arthritis, ΔΔCt equals zero and 2^0 ^equals one. For wild-type and knockout (KO) samples with arthritis, the value of 2^-ΔΔCt ^indicates the fold change in gene expression relative to the wild-type and knockout controls, respectively. Melting curves and agarose gel electrophoresis established the purity of the amplified band.

### Determination of cytokines in mice arthritic knees

Knee joints were obtained, frozen in liquid nitrogen and homogenized in 0.5 ml ice-cold 20 mM Hepes buffer supplemented with 1 mM dithiothreitol, 0.1% v/v Triton and a protease inhibitor cocktail. After incubation for 30 minutes at 4°C, the homogenate was centrifuged for 10 minutes at 10,000 × *g*. Protein concentration was measured in supernatants by the Bradford method and a volume containing 100 μg of proteins was subjected to ELISA for IL-1β, TNF-α, IL-6 and MCP-1 (OptEIA ELISA Sets, BD Pharmingen), according to the manufacture's instructions.

### Statistical analysis

Differences between experimental groups were assessed by ANCOVA, MANCOVA and Mann-Whitney *U *test. *p *values <0.05 were considered significant.

## Results

### PARP-1 protein expression in joint tissue

**Figure 1:**
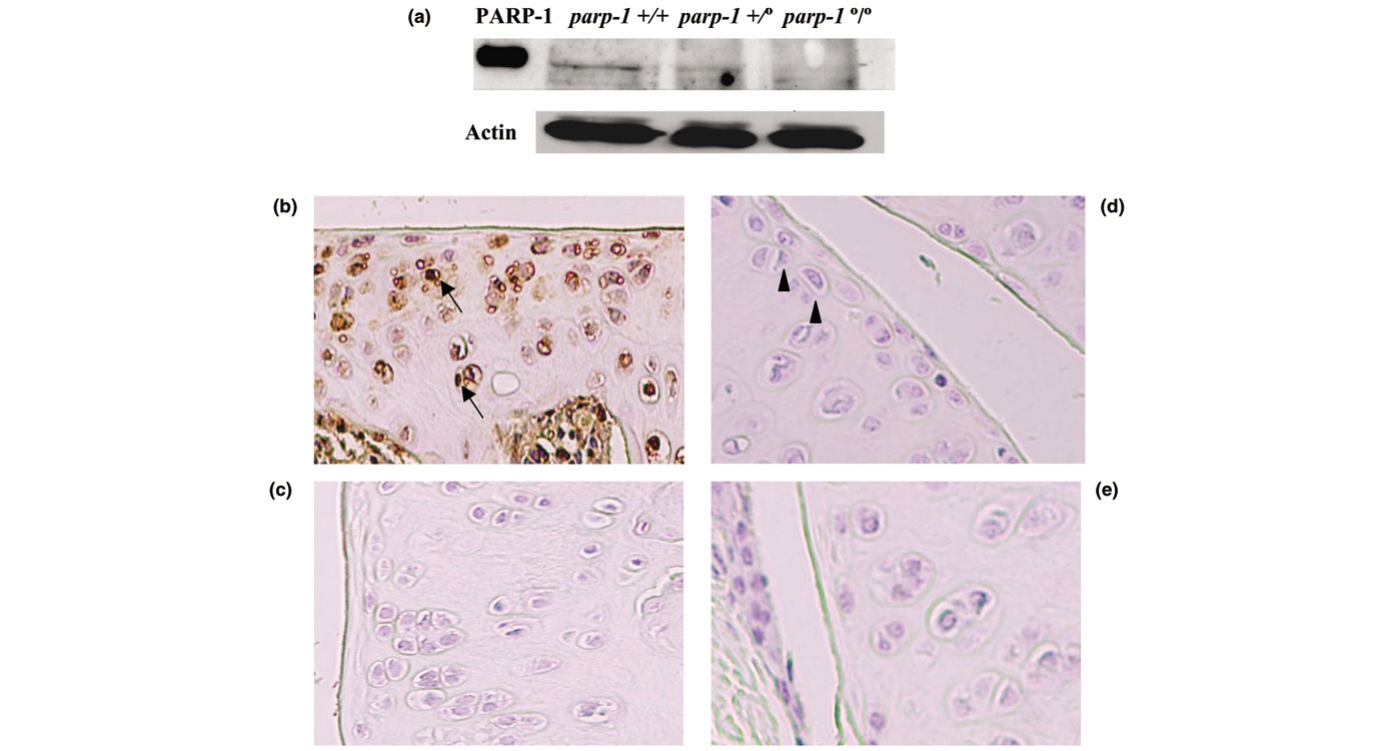
Poly(ADP-ribose) polymerase (PARP)-1 expression in joint tissue. **(a) **Western blot analysis of PARP-1 and actin proteins in fibroblast-like synoviocyte extracts of the indicated genotypes; 100 ng of purified PARP-1 was the control. Immunostaining for PARP-1 expression in the knee joint sections from **(b) ***parp-1*^+^and **(d) ***parp-1*^o/o ^mice. Staining for nuclear PARP-1 showed clear immunopositivity on chondrocytes from *parp-1*^+ ^mice (arrows) whereas chondrocytes from *parp-1*^o/o ^(arrowheads) remained negative. Negative control staining, by omitting the primary antibody, in knee joints from **(c) ***parp-1*^+ ^and **(e) ***parp-1*^o/o ^mice.

Although PARP-1 is found in the majority of the nucleated cells of the body, its expression in joint tissue has never been studied. Here, we have analyzed PARP-1 protein expression by western blot in isolated FLSs from wild-type (*parp-1*^+/+^) mice, mice lacking PARP-1 (*parp-1*^o/o^), and *parp-1 *heterozygous mice (*parp-1*^+/o^). PARP-1 was highly expressed in FLSs from PARP-1 wild-type mice, moderately expressed in *parp-1*^+/o ^and was absent in *parp-1*^o/o ^mice (Figure 1a). Comparable results were obtained in the immunohistochemical analysis of joint sections from *parp-1*^+/+ ^and *parp-1*^o/o ^mice with anti-PARP-1 antibody (Figure 1b, d).

### Reduced severity of arthritis in mice lacking PARP-1

**Figure 2:**
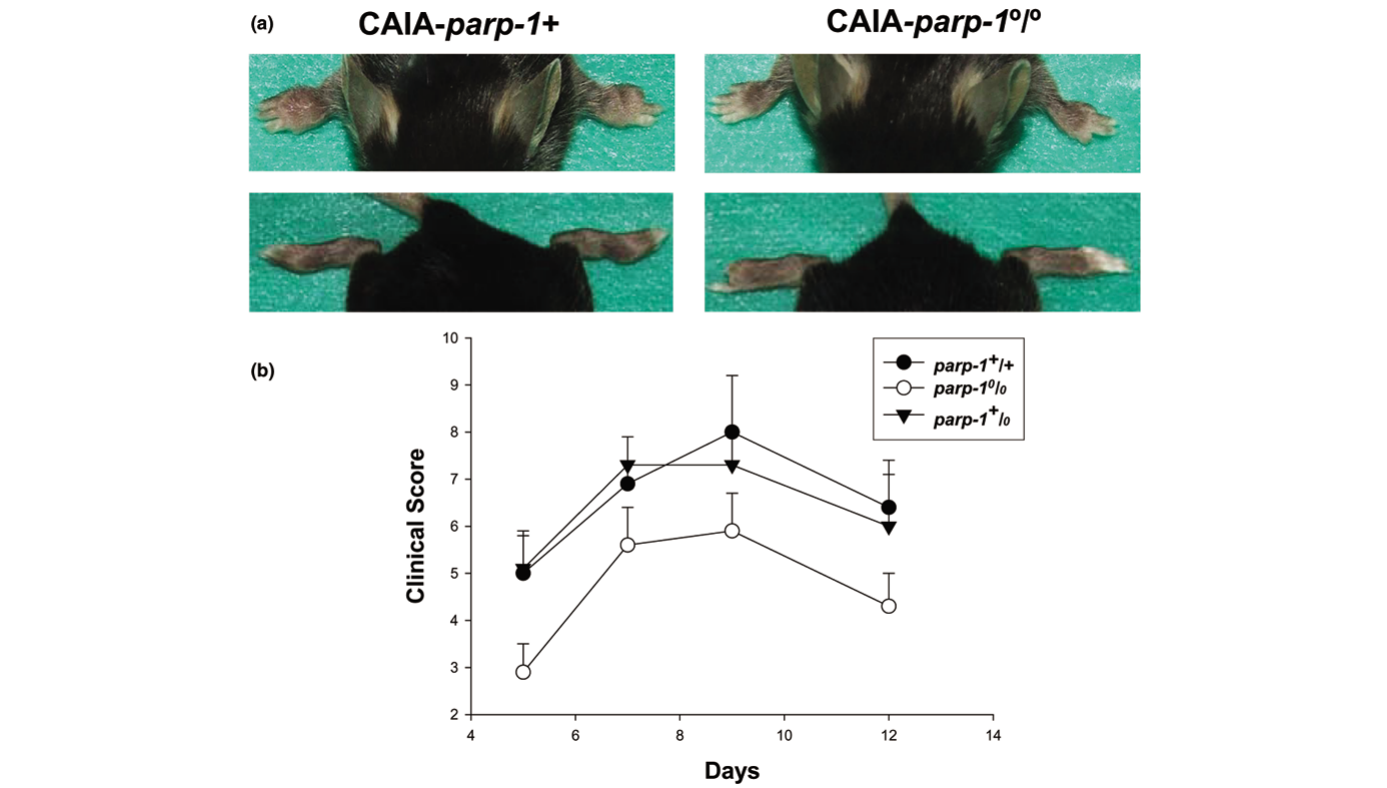
Reduced severity of arthritis in poly(ADP-ribose) polymerase (PARP)-1 deficient mice and PARP-1 sufficient mice following collagen antibody-induced arthritis induction. **(a) **Representative pictures of arthritis in the *parp-1*^+ ^(left panels) and *parp-1*^o/o ^(right panels) mice. **(b) **Clinical score was measured in 18 *parp-1*^+/+^, 8 *parp-1*^+/o ^and 19 *parp-1*^o/o ^mice from day 5 to day 12 after injection. Values are expressed as mean ± standard error of the mean; *p *= 0.03, *parp-1*^+/+ ^and *parp-1*^+/o ^versus *parp-1*^o/o ^mice, by ANCOVA test.

To investigate the contribution of PARP-1 to experimental arthritis, we induced CAIA in control and PARP-1 deficient mice (Figure 2a). In eight separate experiments, male and female *parp-1*^+/+ ^(n = 18), *parp-1*^+/o ^(n = 8) and *parp-1*^o/o ^(n = 19) mice were injected with Arthrogen and lipopolysaccharide and monitored for signs of arthritis. Evolution of arthritis was evaluated by two blinded observers on a 0 to 4 scale, as described in Materials and methods.

There was no difference in incidence or clinical course of arthritis in *parp-1*^o/o ^animals compared with *parp-1*^+ ^control mice. The incidence of disease was 100% in control mice and 94.7% in *parp-1*^o/o ^mice. In both groups, arthritis developed rapidly, the signs of disease appearing as soon as three to five days after the injection of antibody, and reached maximum severity around day seven to nine. PARP-1 deficient mice consistently displayed significantly lower severity of arthritis than *parp-1*^+^control mice (*p *= 0.03 by repeated measures 1-way ANCOVA test) all through the follow-up (Figure 2b).

These results suggest that PARP-1 has a role in the pathogenesis of this arthritis model.

As *parp-1*^+/+ ^and *parp-1*^+/o ^control mice had similar clinical phenotypes, for further analysis, they were pooled together and considered as *parp-1*^+^.

### Reduced histological features of joint inflammation and cartilage damage in PARP-1 deficient mice

**Figure 3:**
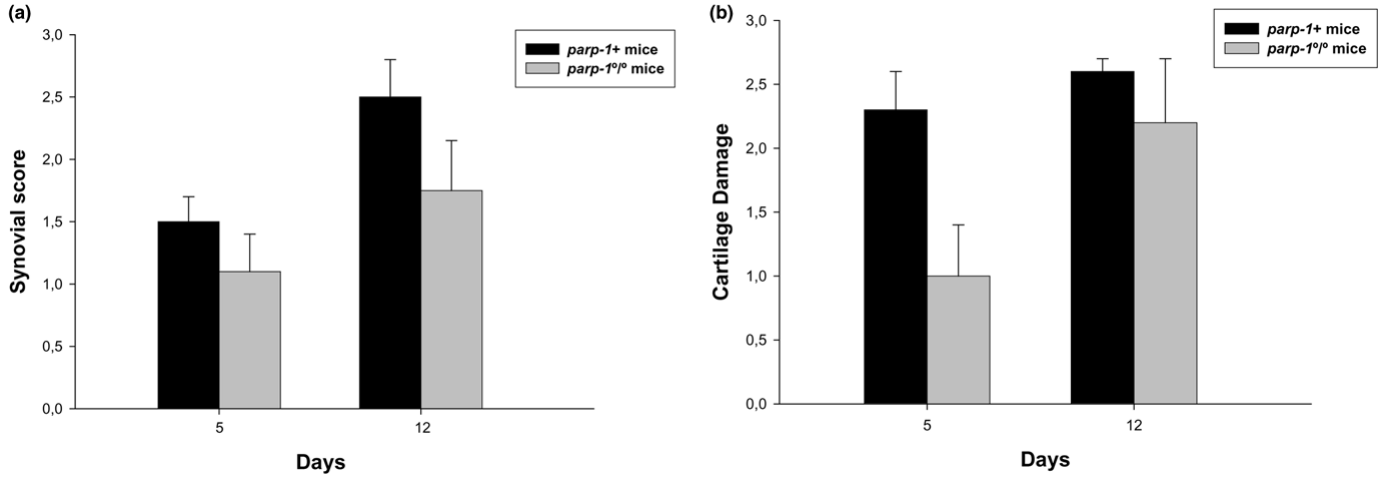
Milder synovial inflammation and cartilage damage in poly(ADP-ribose) polymerase (PARP)-1 deficient mice than in control mice. Histopathological scoring of **(a) **synovial inflammation and **(b) **cartilage damage of knee joint sections of *parp-1*^+^mice and *parp-1*^o/o ^mice at day 5 and 12 after induction of collagen antibody-induced arthritis. Values are expressed as mean ± standard error of the mean; *p *= 0.03, *parp-1*^+ ^versus *parp-1*^o/o ^mice by combined analysis of (a) and (b) (MANCOVA test).

**Figure 4:**
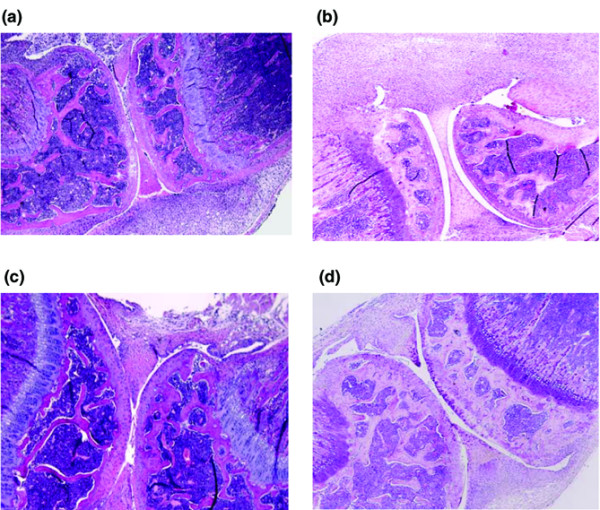
Representative hematoxylin-eosin stained sections of knee joints in mice with collagen antibody-induced arthritis (CAIA). Severe inflammation, pannus formation and associated cartilage destruction were observed in sections stained with hematoxylin-eosin from *parp-1*^+ ^mice at **(a) **day 5 and **(b) **day 12 after CAIA induction compared to *parp-1*^o/o ^mice at **(c) **day 5 and **(d) **day 12.

To quantify joint involvement, we assessed synovial inflammation in H&E stained sections of knee joints. Joints were taken from 14 *parp-1*^o/o ^and 13 *parp-1*^+ ^mice on days 5, 7 and 12, and histological sections were scored by two blinded observers on a 0 to 3 scale, corresponding to the degree of thickening of the synovial lining, sublining infiltration and pannus formation. On this scale, we observed a clear trend to a lower synovial inflammation score in *parp-1*^o/o ^mice compared to *parp-1*^+^mice (Figures 3 and 4), although the difference was not significant (*p *= 0.058, by 1-way MANCOVA fixed effects test).

**Figure 5:**
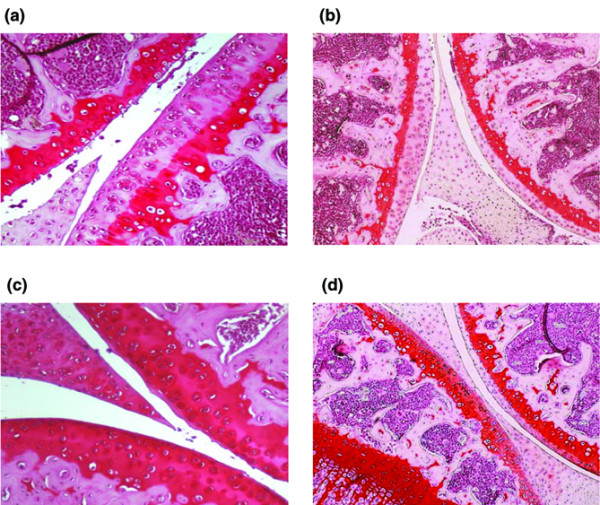
Representative sections stained with Safranin O in mice with collagen antibody-induced arthritis (CAIA). More severe loss of proteoglycans, indicated by destained cartilage layers, were observed in sections from *parp-1*^+ ^mice at **(a) **day 5 and **(b) **day 12 after CAIA induction compared with knockout mice at **(c) **day 5 and **(d) **day 12.

Joint sections were also stained with Toluidine blue, Safranin-O and Masson trichrome to evaluate cartilage damage. The results also showed a trend to less damage in *parp-1*^o/o ^mice (Figures 3 and 5), although, again, the difference did not reach statistical significance (*p *= 0.053, by 1-way MANCOVA fixed effects test). When we considered synovial inflammation and cartilage damage jointly as two facets of the arthritic lesions, the difference between *parp-1*^o/o ^and *parp-1*^+ ^mice was significant (*p *= 0.03).

Thus, PARP-1 protein appeared to be involved in the pathogenesis of the CAIA model, both in synovial inflammation and cartilage damage, although it did not seem to have a pivotal role.

**Figure 6:**
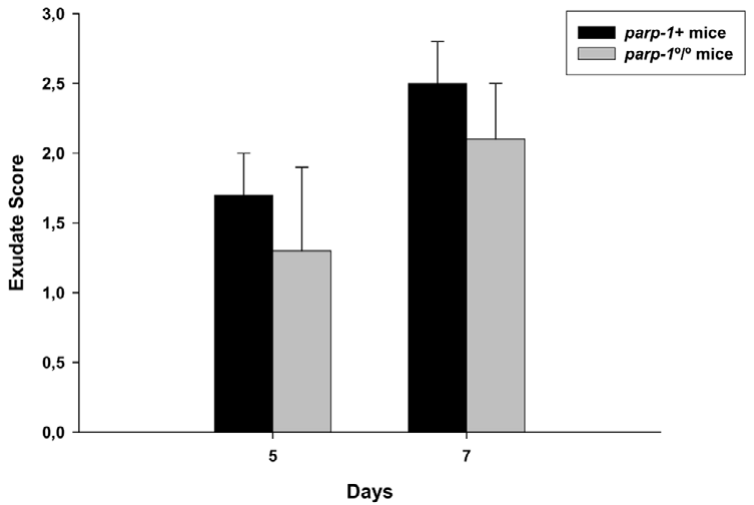
Comparable exudate scores in the arthritic joints of *parp-1*^+ ^and *parp-1*^o/o ^mice. Exudate score was evaluated in knee sections stained with hematoxylin-eosin from *parp-1*^+^and *parp-1*^o/o ^mice at day 5 and 7 after induction of collagen antibody-induced arthritis. Values represent the mean ± standard error of the mean. Differences between *parp-1*^+^and *parp-1*^o/o ^mice were not statistically significant (*p *= 0.12 by 1-way MANCOVA test).

It has been previously described that, in several rodent models of inflammation, PARP-1 activation is involved in neutrophil recruitment [25]. Neutrophils have been implicated in arthritis disease; specifically, extensive neutrophil exudate is displayed in CAIA model [23] and neutrophils release elastase and proteases, which degrade proteoglycans [4]. It remains possible that the decreased severity of arthritis observed in PARP-1 deficient mice was associated with reduced neutrophil exudate in joints from these mice. To evaluate this possibility, we assessed, at five and seven days, the exudate score on a 0 to 3 scale in H&E stained sections of knee joints. Exudate appeared slightly lower in *parp-1*^o/o ^compared to *parp-1*^+ ^mice, although the difference was not significant (*p *= 0.12, by 1-way MANCOVA fixed effects test) (Figure 6). Thus, lack of PARP-1 does not impair neutrophil exudation in this arthritis model.

### Expression levels of inflammatory mediators in arthritic joints from PARP-1 deficient and sufficient mice

**Figure 7:**
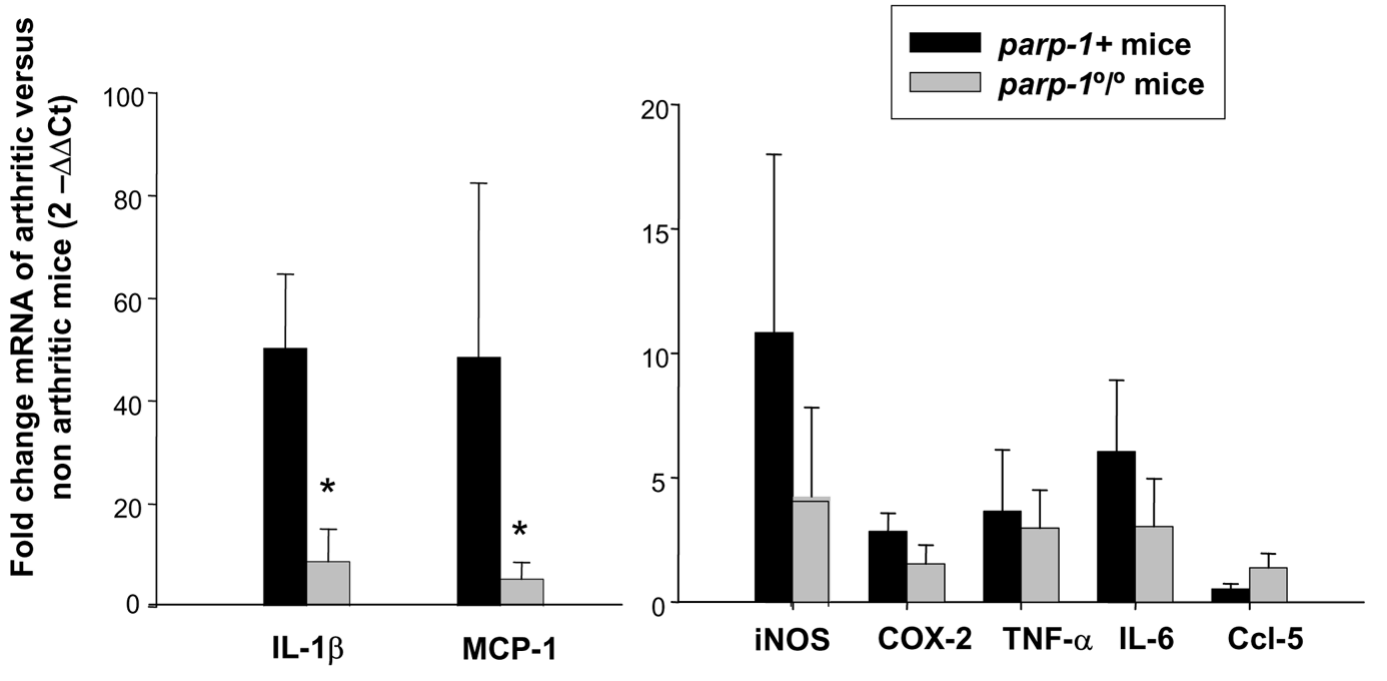
Reduced IL-1β and monocyte chemotactic protein (MCP)-1 mRNA levels in mice lacking poly(ADP-ribose) polymerase (PARP)-1. IL-1β, MCP-1, inducible nitric oxide synthase (iNOS), cyclooxygenase (COX)-2, tumor necrosis factor (TNF)-α, IL-6 and small inducible cytokine A5 (Ccl5) mRNA levels were measured by quantitative real-time PCR in arthritic joints of *parp-1*^+ ^and *parp-1*^o/o ^mice at day 7 after induction of collagen antibody-induced arthritis. Values are expressed as mean ± standard error of the mean of six to nine mice per group. Differences between *parp-1*^+ ^and *parp-1*^o/o ^mice were statistically significant for IL-1β (asterisk indicates *p *= 0.005) and MCP-1 (asterisk indicates *p *= 0.004) by Mann-Whitney *U *test.

To explore the possible mechanisms underlying the reduced arthritis observed in PARP-1 knockout mice compared to control mice, we studied, by quantitative real-time PCR, mRNA levels of IL-1β, IL-6, TNF-α, MCP-1, small inducible cytokine A5 (Cc15; RANTES), inducible nitric oxide synthase (iNOS) and cyclooxygenase (COX)-2 in arthritic joints at day seven after Arthrogen injection. The fold change in mRNA of arthritic versus non-arthritic *parp-1*^o/o ^and *parp-1*^+ ^mice is shown in Figure 7. All the inflammatory mediators were detected in both groups of mice and, interestingly, IL-1β and MCP-1 mRNA were significantly less induced in arthritic *parp-1*^o/o ^compared to arthritic *parp-1*^+ ^mice. IL-6 mRNA showed a trend towards lower induction in *parp-1*^o/o ^compared to *parp-1*^+ ^arthritic mice (*p *= 0.1, by Mann-Whitney *U *test). However, mRNA expression of TNF-α and Ccl5 were induced to a similar extend in both groups of arthritic mice (*p *= 0.9 and *p *= 0.7, respectively, by Mann-Whitney *U *test). Transcription of genes encoding iNOS and COX-2, which are involved in the synthesis of nitric oxide and prostaglandin E_2_, respectively, were induced to levels that were not significantly different in PARP-1 deficient and sufficient arthritic mice. However, a tendency towards lower induction in the *parp-1*^o/o ^mice was noted.

**Figure 8:**
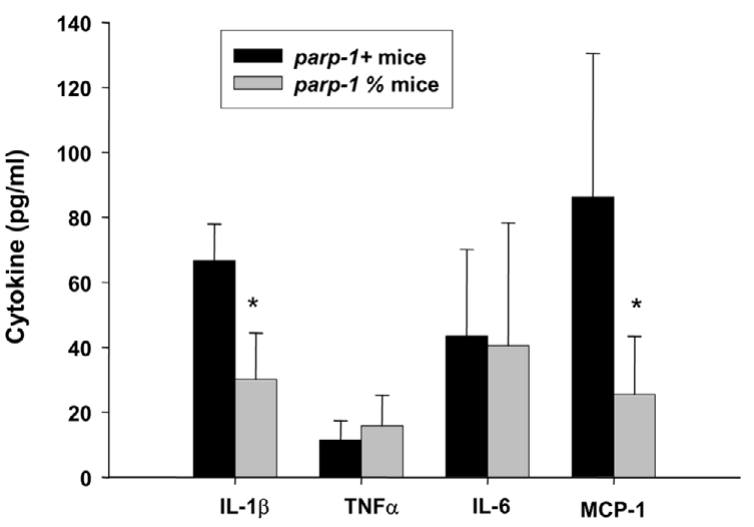
Reduced IL-1β and monocyte chemotactic protein (MCP)-1 levels in arthritic joints of mice lacking poly(ADP-ribose) polymerase (PARP)-1. Levels of IL-1β, tumor necrosis factor (TNF)-α, IL-6 and MCP-1 were measured by ELISA in extracts from arthritic knee joints of mice at day 7 after induction of collagen antibody-induced arthritis. Values are expressed as mean ± standard error of the mean of six to nine mice per group. Differences between *parp-1*^+ ^and *parp-1*^o/o ^mice were statistically significant for IL-1β (asterisk indicates *p *= 0.03) and MCP-1 (asterisk indicates *p *= 0.05) by Mann-Whitney *U *test.

To confirm these findings, we next determined the levels of IL-1β, IL-6, TNF-α and MCP-1 proteins in joint tissues. IL-1β and MCP-1 were significantly reduced in joints from arthritic *parp-1*^o/o ^compared to arthritic *parp-1*^+ ^mice (Figure 8); however, there was no difference in the production of TNF-α and IL-6 in both groups of mice (*p *= 0.4 and *p *= 0.3, respectively, by Mann-Whitney *U *test). These results are consistent with those obtained for the mRNA analysis.

## Discussion

Previous studies using genetically engineered animals and pharmacological inhibitors have implicated PARP-1 in the pathogenesis of several inflammatory processes [13-16]. In the present report, we have investigated the impact of selective PARP-1 suppression in the CAIA model and found that absence of PARP-1 protein reduces the severity of disease, likely by the impairment of IL-1β and MCP-1 transcription in joint tissues. In the arthritis model used, disease develops in most mice strains, avoiding multiple breeding into arthritis susceptible strains. Using a suitable antibody dose, arthritis incidence rises to 100% in control animals and the clinical severity and histopathology are similar to collagen-induced arthritis and human RA. Given the involvement of PARP-1 in inflammation, we considered that it could have a role in the effector phase of arthritis; the CAIA model specifically reflects this phase.

PARP-1 deficient mice had decreased severity in clinical and histological arthritis, although the incidence of disease was similar in control and deficient mice. This is in line with its described involvement in other inflammatory diseases, but with milder effect in the case of arthritis.

PARP-1 belongs to a large family of 18 proteins, encoded by different genes and displaying a conserved catalytic domain (for reviews, see [26,27]). PARP-1 catalyzes 80% of cellular poly(ADPribosyl)ation and the other PARP family members, PARP-2, PARP-3, PARP-4 and Tankyrases (PARP-5 a and b), all identified in the last few years, account for the remaining 20%. Therefore, it is possible that when PARP-1 is absent from development, other PARP family members with poly(ADPribosyl)ation activity could compensate for its absence. To evaluate this possibility, we treated *parp-1*^o/o ^and *parp-1*^+ ^mice with 3,4-dihydro-5- [4-(1-piperidinyl)butoxy]-1(2*H*)-isoquinolinone (DPQ), one of the new potent PARP inhibitors developed. After treatment, we found similar protection to that observed in mice lacking the *parp-1 *gene (data not shown), suggesting that PARP-1 is the member of the PARP proteins involved in arthritis inflammation.

Our results contrast with the significantly reduced incidence and severity of collagen induced arthritis in mice treated with INH_2_BP, a PARP inhibitor, reported by Szabo and colleagues [28]. It is possible that INH_2_BP has effects other than the inhibition of PARP function, because we did not observe such a strong effect with knockout mice, nor with the DPQ inhibitor. Nevertheless, this discordance could also be attributed to either differences in the arthritis model or differences in the inhibitors. In fact, it has been recently shown that another PARP inhibitor, PJ34, reduces the severity rather than incidence of collagen induced arthritis [16].

IL-1β is one of the major cytokines in arthritis driving inflammation and joint destruction [2,4,7]. It has been reported that systemic administration of IL-1 accelerates and exacerbates the development of murine collagen induced arthritis [29], while IL-1 receptor antagonist-deficient mice (BALB/c background) develop chronic polyarthropathy resembling RA [30]. MCP-1 is a potent chemoattractant for monocytes. It seems to be involved in RA pathogenesis because it has been detected in patient sera and found at increased levels in FLSs from RA patients [31-33]. It has been recently reported that MCP-1 induces FLS proliferation, which is pivotal in pannus formation, and increases metalloproteinase production mediated by IL-1β [34].

Thus, the strong reduction in IL-1β and MCP-1 production observed in PARP-1 deficient mice may account for the reduced severity of arthritis, though signals of a more widespread effect are reflected in the tendency towards the decreased expression of other inflammatory mediators, such as IL-6, iNOS and COX-2.

In contrast to what has been described in the shock endotoxic model [13,16], we did not find impaired TNF-α production in mice lacking PARP-1. This could indicate that the requirements of PARP-1 for transcription of inflammatory genes depend on the tissue and the nature of the inflammatory stimulus. In fact, studies with a PARP inhibitor have shown an inhibitory or neutral effect on IL-1β levels depending on the model of inflammation [16].

## Conclusion

Overall, our results indicate that PARP-1 plays a role in arthritis progression, probably through impaired IL-1β and MCP-1 production in joints. Although further investigations are required to evaluate PARP-1 involvement in human RA, this enzyme might be considered as a new target for experimental treatment.

## Abbreviations

CAIA = collagen antibody-induced arthritis; Ccl5 (RANTES) = small inducible cytokine A5; COX-2 = cyclooxygenase; DMEM = Dulbecco's modified Eagle's medium; DPQ = 3,4-dihydro-5- [4-(1-piperidinyl)butoxy]-1(2*H*)-isoquinolinone; FLS = fibroblast-like synoviocyte; H&E = hematoxylin and eosin; IL = interleukin; iNOS = inducible nitric oxide synthase; MCP = monocyte chemotactic protein; NF = nuclear factor; PARP = poly(ADP-ribose) polymerase; RA = rheumatoid arthritis; TNF = tumor necrosis factor.

## Competing interests

The authors declare that they have not competing interests.

## Authors' contributions

SG carried out the arthritis evolution, FLS isolation and western blot experiments and quantitative real time PCR analysis. AB carried out the breeding of mice, the arthritis evolution experiments and joint isolation. AG carried out the intravenous injections in mice, performed the statistical analysis, participated in the design of the study and revision of the manuscript. JF carried out the histological scoring. GJ participated in the design and coordination of the study and revision of the manuscript. CC conceived of the study, participated in its design and coordination and drafting of the manuscript. All authors read and approved the final manuscript.

## Figures and Tables

**Table 1:** Primer sets used for quantitative PCR study

Gene	Forward primer	Reverse primer
*IL-1β*	AACCTGCTGGTGTGTGACGTTC	CAGCACGAGGCTTTTTTGTTGT
*TNF-α*	CTACTCCCAGGTTCTCTTCAA	GCAGAGAGGAGGTTGACTTTC
*IL-6*	ACAACCACGGCCTTCCCTACTT	CACGATTTCCCAGAGAACATGTG
*MCP-1*	CCACTCACCTGCTGCTACTCAT	TGGTGATCCTCTTGTAGCCCTCC
*Ccl5*	GTCGTGTTTGTCACTCGAAGGA	TTGATGTATTCTTGAACCCACTTCTT
*iNOS*	CAGCTGGGCTGTACAAACCTT	CATTGGAAGTGAAGCGTTTCG
*COX-2*	GTGGAAAAACCTCGTCCAGA	GCTCGGCTTCCAGTATTGAG
β*-Actin*	AGGTCATCACTATTGGCAACGA	CACTTCATGATGGAATTGAATGTAGTT
